# The Notch Signaling Pathway Is Balancing Type 1 Innate Lymphoid Cell Immune Functions

**DOI:** 10.3389/fimmu.2018.01252

**Published:** 2018-06-07

**Authors:** Thibaut Perchet, Maxime Petit, Elena-Gaia Banchi, Sylvain Meunier, Ana Cumano, Rachel Golub

**Affiliations:** ^1^Unit for Lymphopoiesis, Department of Immunology, Pasteur Institute, Paris, France; ^2^INSERM U1223, Paris, France; ^3^Université Paris Diderot, Sorbonne Paris Cité, Cellule Pasteur, Paris, France

**Keywords:** Notch, innate lymphoid cells, liver, cancer, inflammation, transcription factors, cytotoxicity, molecular biology techniques

## Abstract

The Notch pathway is one of the canonical signaling pathways implicated in the development of various solid tumors. During carcinogenesis, the Notch pathway dysregulation induces tumor expression of Notch receptor ligands participating to escape the immune surveillance. The Notch pathway conditions both the development and the functional regulation of lymphoid subsets. Its importance on T cell subset polarization has been documented contrary to its action on innate lymphoid cells (ILC). We aim to analyze the effect of the Notch pathway on type 1 ILC polarization and functions after disruption of the RBPJk-dependent Notch signaling cascade. Indeed, type 1 ILC comprises conventional NK (cNK) cells and type 1 helper innate lymphoid cells (ILC1) that share Notch-related functional characteristics such as the IFNg secretion downstream of T-bet expression. cNK cells have strong antitumor properties. However, data are controversial concerning ILC1 functions during carcinogenesis with models showing antitumoral capacities and others reporting ILC1 inability to control tumor growth. Using various mouse models of Notch signaling pathway depletion, we analyze the effects of its absence on type 1 ILC differentiation and cytotoxic functions. We also provide clues into its role in the maintenance of immune homeostasis in tissues. We show that modulating the Notch pathway is not only acting on tumor-specific T cell activity but also on ILC immune subset functions. Hence, our study uncovers the intrinsic Notch signaling pathway in ILC1/cNK populations and their response in case of abnormal Notch ligand expression. This study help evaluating the possible side effects mediated by immune cells different from T cells, in case of multivalent forms of the Notch receptor ligand delta 1 treatments. In definitive, it should help determining the best novel combination of therapeutic strategies in case of solid tumors.

## Introduction

Type 1 innate lymphoid cells (ILC) are defined by the capacity to secrete IFNg and comprise at least two distinct subsets, type 1 helper innate lymphoid cells (ILC1) that are the tissue-resident counterparts of the circulating conventional NK (cNK) cells found in blood and in numerous tissues. ILC1 have been identified in liver, gut, salivary glands, skin, peritoneum, spleen, and uterus (1). cNK and ILC1 both express the receptors NKp46 and NK1.1 that distinguishes them from other ILC subsets. ILC1 express markers of tissue residency with an immature CD49a^+^CD49b^−^ phenotype in the liver. cNK and ILC1s could be discriminated by the identity of T-box transcription factors they expressed. The T-box protein in T cells, T-bet, is encoded by the *Tbx21* gene involved in IFNg production. Another T-box transcription factor eomesodermin (Eomes) shares homology with T-bet. Mature cNK cells are T-bet^+^ Eomes^+^ and T-bet upregulation is induced during ILC differentiation in the liver. Studies in Eomes reporter mice showed that despite their immature phenotype, T-bet^+^ hepatic ILC1 are not precursors of Eomes^+^ cNK cells (2) and ILC1 and cNK lineages diverge early in ontogeny (3). There is limited information on the mechanisms inducing or repressing T-box transcription factors in different organs. Because *Tbx21* is a known potential target of the Notch canonical pathway, we investigated the role of the Notch signaling pathway on the differentiation and function of ILC1 and cNK cells residing in enterohepatic sites. The Notch pathway is highly conserved and regulates several aspects of development and differentiation (4, 5). Vertebrates express four different Notch receptors (Notch 1–4), which can engage five known ligands (Delta-like 1, 3, 4, Jagged 1, and 2) (6). The co-factor RBP-Jk and mastermind-like (MAML) mediate the signaling cascade of the canonical Notch pathway. Upon recognition of Notch ligands and after serial proteolytic cleavages, the Notch intracellular domain translocates to the nucleus where it binds to the transcription factor CSL/RBP-Jk, recruiting co-activator members of the MAML family, and enabling transcription of target genes (6, 7). Notch receptors are expressed in the hematopoietic cells with a well-known role of Notch1 signaling in regulating T versus B cell fate decisions (8). *In vitro* studies have shown that multipotent progenitors differentiate into T/cNK progenitors *via* the Notch1/Dll1 or Dll4 interaction; however, at later stages, Notch1 favor the T cell potential to the expense of cNK cells (9–11). It has been proposed that the Notch ligand Jagged2 promotes the development of cNK from murine hematopoietic progenitors (12). At later stages, Notch signaling has been implicated in the upregulation of KIR molecules (13) and in human peripheral cNK cells it increases IFNg secretion (14). cNK cells participate to immune surveillance of tumors and viral infection (15, 16). They are important cytotoxic players by the release of granules containing both perforin and granzyme B (GzmB) and by the production of inflammatory cytokines, such as IFNg and TNFa.

There is, however, limited information for a role of Notch pathway in ILC1 development and function. Single-cell transcriptional analyses of hepatic ILC1 and cNK showed that more than half of both cell types express Notch receptors. Moreover, gene expression analysis indicated a possible implication of the Notch signaling pathway on the heterogeneity of these populations. We therefore analyzed ILC1 in mice where the canonical Notch pathway is abrogated in all lymphoid lineages using a conditional knockout of RBP-Jk in cells expressing IL-7Ra, a receptor upregulated at the common lymphoid progenitor stage (11, 17). We found that both cNK and ILC1 are altered in the absence of the Notch signaling pathway. Hepatic and circulating ILC1 from Il7r^Cre^ Rbpj^F/F^ mice showed decreased expression of CD49a and the ratio of cNK versus ILC1 were affected possibly due to a deregulation of proliferation/survival.

Considering that the Notch pathway activates Th1 type responses, we expected that the lack of Notch signaling would reduce inflammatory responses in type 1 ILC. Instead, we found that RBPJ deficiency enhanced the inflammatory and cytotoxic functions of the type 1 ILC subsets present in the enterohepatic region. Notably, we showed that T-bet was inhibited in RBPJ-deficient cells resulting in the upregulation of the complementary Eomes and of an inflammatory gene signature. Finally, we showed that RBPJ deficiency also increased the control of tumor proliferation at the early time-points due to the recruitment of highly inflammatory cNK cells. We conclude that Notch signaling, in type 1 ILC cells, prevents the over-expression of pro-inflammatory cytokines through the regulation of T-bet and Eomes expression.

## Materials and Methods

### Mice

IL7r^+/+^, IL7r^Cre/+^ Rbpj^F/+^, IL7r^Cre/+^ Rbpj^F/F^, Vav^Cre/+^ Rbpj^F/+^, Vav^Cre/+^ Rbpj^F/F^, IL7r^Cre/+^ Notch2^F/+^, and IL7r^Cre/+^ Notch2^F/F^ mice were bred in the animal facilities at Pasteur Institute, Paris. Mice were bred in accordance with Pasteur Institute guidelines in compliance with European animal welfare regulations, and all animal studies were approved by Pasteur Institute Safety Committee in accordance with French and European guidelines.

### Cell Preparation

Bone marrow (BM), thymic lobes, spleens, and lamina propria lymphocytes (LPL) were harvested, dissociated, and resuspended in Hanks’ balanced salt solution (HBSS) supplemented with 1% fetal calf serum (FCS; Gibco). To isolate LPL, the small bowel was flushed with phosphate-buffered saline (PBS), and the conjunctive tissue and Peyer’s patches were carefully removed. The intestine was opened and cut into 1-cm pieces. To eliminate epithelial cells and intraepithelial lymphocytes, these fragments were incubated at 37°C in 50 ml of RPMI 1640 (Gibco) containing 10% FCS and 10 mM Hepes buffer under strong agitation for 30 min, which was followed by vortex treatment for 4 min. For LPL isolation, the remaining fragments were incubated in identical medium to which was added type VIII collagenase (0.5 mg/ml; Sigma-Aldrich) and were shaken for 30 min at 37°C. To complete digestion, the suspension was repeatedly passed through a 10-ml syringe for 5 min and then filtered through a 40-mm cell strainer (BD Biosciences) and collected by centrifugation. The cell pellet was resuspended in 44% Percoll (GE Healthcare), laid over 67% Percoll, and centrifuged at 600 *g* for 20 min at 20°C. Cells at the interface were collected, washed in HBSS containing 1% FCS, and recovered. Livers were harvested, dissociated, and resuspended in RPMI 1640 supplemented with 2% FCS. Cells were collected by centrifugation and resuspended in 44% Percoll. After centrifugation at 600 *g* for 20 min at 20°C, the cell pellet was washed in HBSS containing 1% FCS. Blood and portal vein blood (PVB) were harvested using a 1-ml syringe (BD Plastipak) and laid on Ficoll Paque Plus (GE Healthcare). After centrifugation at 600 *g* for 20 min at 20°C, the cell at the interface were washed in HBSS containing 1% FCS and recovered. Tumors were harvested and then washed with PBS, then separated into different tubes. The tumors were resuspended in 2 ml of thermolysin (Liberase™, Roche Diagnostics, Mannheim, Germany) at 0.13 U/ml (concentrations as recommended by the manufacturer). Incubations were performed for 30 min at 37°C. Following incubation, the digestate was crushed and passed through a 70-µm filter and washed with RPMI supplemented with 10% FCS. Samples were centrifuged at 370 *g* for 7 min and resuspended in HBSS containing 1% FCS.

### Flow Cytometry

Flow cytometry data were acquired with a LSRFortessa flow cytometer (Becton Dickinson) and analyzed with FlowJo software (Tree Star). Dead cells were eliminated by exclusion with propidium iodide. Cells were stained intracellularly after permeabilization and fixation with True Nuclear Transcription Factor Buffer Set (BioLegend). Cells were purified with a FACSAria III (Becton Dickinson) and recovered in tubes or in 96-well quantitative PCR (qPCR) plates for gene expression analysis.

### Antibodies

All antibodies were from BD Biosciences, eBioscience, BioLegend, Cell Signaling Technology, or R&D Systems. Antibodies were biotinylated or conjugated to fluorochromes (fluorescein isothiocyanate, phycoerythrin, PECy5, PerCPCy5.5, PECy7, allophycocyanin, Alexa Fluor 647, APCCy7, Pacific Blue, BV421, eFluor450, V500, BV605, BV655, BV700, and BV786) and were specific for the following mouse antigens: Ly76 (TER119), Gr-1 (RB6-8C5), CD3e (145-2C11), CD19 (6D5), NK1.1 (PK136), IL-7Ra (A7R34), CD8 (53-6.7), TCRb (H57-597), TCRd (GL3), CD4 (GK1.5), Thy1.2 (53-2.1), NKp46 (29A1.4), IFNg (XMG1.2), CD27 (LG.3A10), CD45.2 (104), CD49a (HMa1), CD49b (DX5), Eomes (Dan11mag), TNFa (MP6-XT22), PD1 (29F.1A12), CD226 (10E5), Mac1 (M1/70), and GzmB (GB12).

### RT-qPCR Analysis

Cells were sorted in Buffer RLT (Qiagen) containing 2-mercaptoethanol (Sigma-Aldrich) and were frozen at −80°C. RNA was obtained with an RNeasy Micro Kit (Qiagen), and complementary DNA (cDNA) was obtained with the PrimeScript RT Reagent Kit (Takara). A 7300 Real-Time PCR System (Applied Biosystems) and TaqMan technology (Applied Biosystems) or SYBR Green Technology (Qiagen) were used for qRT-PCR analysis. A bilateral unpaired Student’s *t*-test was used for statistical analysis. The following primers were from SABiosciences: Ifng (Mm_01168134_m1), Eomes (Mm_01351984_m1), Tnfa (Mm_00443258_m1), GzmB (Mm_00442837_m1), Hprt (Mm_00446968_m1), and Actb (Mm_02619580_g1).

### Tumor Injection

Cancer Hepa 1.6 cells were cultured in Opti-MEM with GlutaMAX (Gibco) containing 10% FCS (Gibco), 1% penicillin–streptomycin (Gibco), and 60 mM 2-mercaptoethanol (Sigma-Aldrich) and were maintained in a 37°C incubator (Thermo Scientific) with 5% CO_2_. Cells were harvested, washed, and resuspended in PBS. 3 × 10^6^ cells were injected subcutaneously in 150 µl of PBS. Mice were monitored every day and tumor growth was measured every 2/3 days.

### T Cell Transfer

CD3 cells were isolated using magnetic microbead (Miltneyi Biotech, Bergisch Gladbach, Germany) from spleen of B6 wild-type mice. 3 × 10^6^ purified CD3 positive cells were injected in 150 µl of PBS in host mice 3 days before Hepa1.6 injection.

### Cytotoxicity Assay

Freshly isolated splenic cNK cells (Lin^−^ CD45^+^ CD4^−^ NKp46^+^ NK1.1^+^) were sorted to high purity (>98%) and used as effectors. Killing of the cNK-sensitive YAC-1 (European Collection of Cell Cultures) target cells was assessed using a fixable viability dye. Percentage specific of killing was calculated as: 100 × (experimental release − spontaneous release)/(total release − spontaneous release).

### Single Cell Multiplex RT-qPCR

Cells were sorted in 96-well qPCR plates in 9 µl of a CellsDirect One-Step quantitative RT-PCR Kit (Life Technologies), containing mixtures of diluted primers (0.05× final concentration). Preamplified cDNA was obtained after reverse transcription (15 min at 40°C, 15 min at 50°C and 15 min at 60°C), and preamplification (22 cycles: 15 s at 95°C and 4 min at 60°C), and diluted 1:5 in TE pH8 Buffer (Ambion). Sample mix was as follows: diluted cDNA (2.9 µl), Sample Loading Reagent (0.29 µl; Fluidigm), TaqMan Universal PCR Master Mix (3.3 µl; Applied Biosystem), or Solaris quantitative PCR Low ROX Master Mix (3.3 µl; GE Dharmacon). The assay mix was as follows: Assay Loading Reagent (2.5 µl; Fluidigm) and TaqMan (2.5 µl; Applied Biosystem). A 48.48 dynamic array integrated fluidic circuit (IFC; Fluidigm) was primed with control line fluid, and the chip was loaded with assays (TaqMan) and samples using an HX IFC controller (Fluidigm). The experiments were run on a Biomark (Fluidigm) for amplification and detection (2 min at 50°C, 10 min for TaqMan reagents or 15 min for Solaris reagents at 95°C, 40 cycles: 15 s at 95°C and 60 s at 60°C).

### Bioinformatic Analyses

For visualization, the dimensionality of the datasets was further reduced using the “Barnes-hut” approximate version of t-SNE. This was implemented using the Rtsne function from the Rtsne R package using 800 iterations and a perplexity setting that varied from 10 to 30 depending on the size of the dataset. PhenoGraph takes as input a matrix of N single-cell measurements and partitions them into subpopulations by clustering a graph that represents their phenotypic similarity. PhenoGraph builds this graph in two steps. First, it finds the k nearest neighbors for each cell (using Euclidean distance), resulting in N sets of k-neighborhoods. Second, it operates on these sets to build a weighted graph such that the weight between nodes scales with the number of neighbors they share. The Louvain community detection method is then used to find a partition of the graph that maximizes modularity. Given a dataset of N d-dimensional vectors, M distinct classes, and a vector providing the class labels for the first L samples, the PhenoGraph classifier assigns labels to the remaining N_L unlabeled vectors. First, a graph is constructed as described above. The classification problem then corresponds to the probability that a random walk originating at unlabeled node x will first reach a labeled node from each of the M classes. This defines an M-dimensional probability distribution for each node x that records its affinity for each class.

### Statistical Analysis

Statistical analysis was performed with the Student’s *t*-test or two-way analysis of variance. The analysis was performed using Prism Software (GraphPad). Statistical significance is represented as follows: *p < 0.05, **p < 0.01, and ***p < 0.001.

## Results

### Four Distinct Populations of Type 1 ILC Are Defined in the Liver

**Figure 1:**
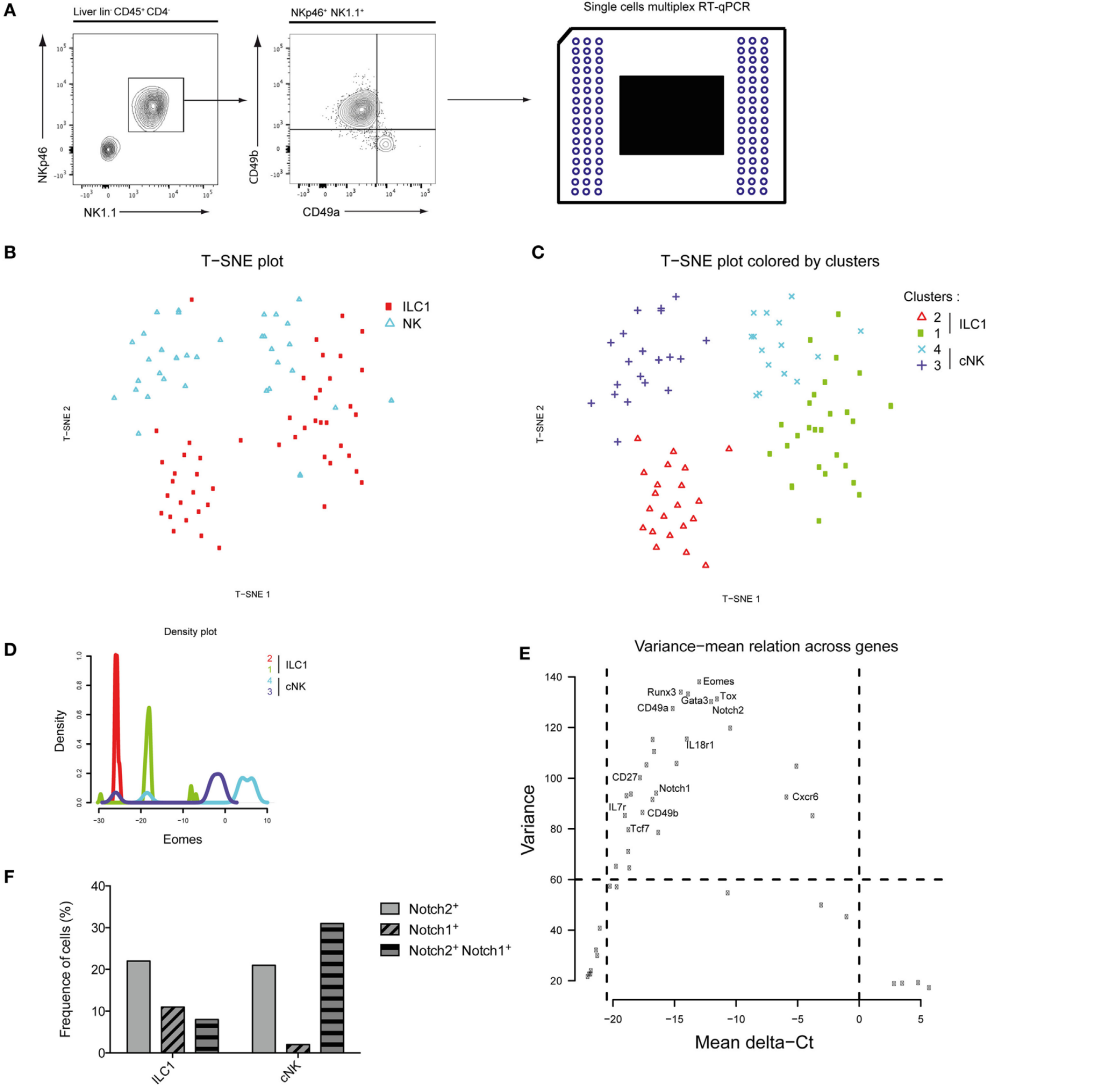

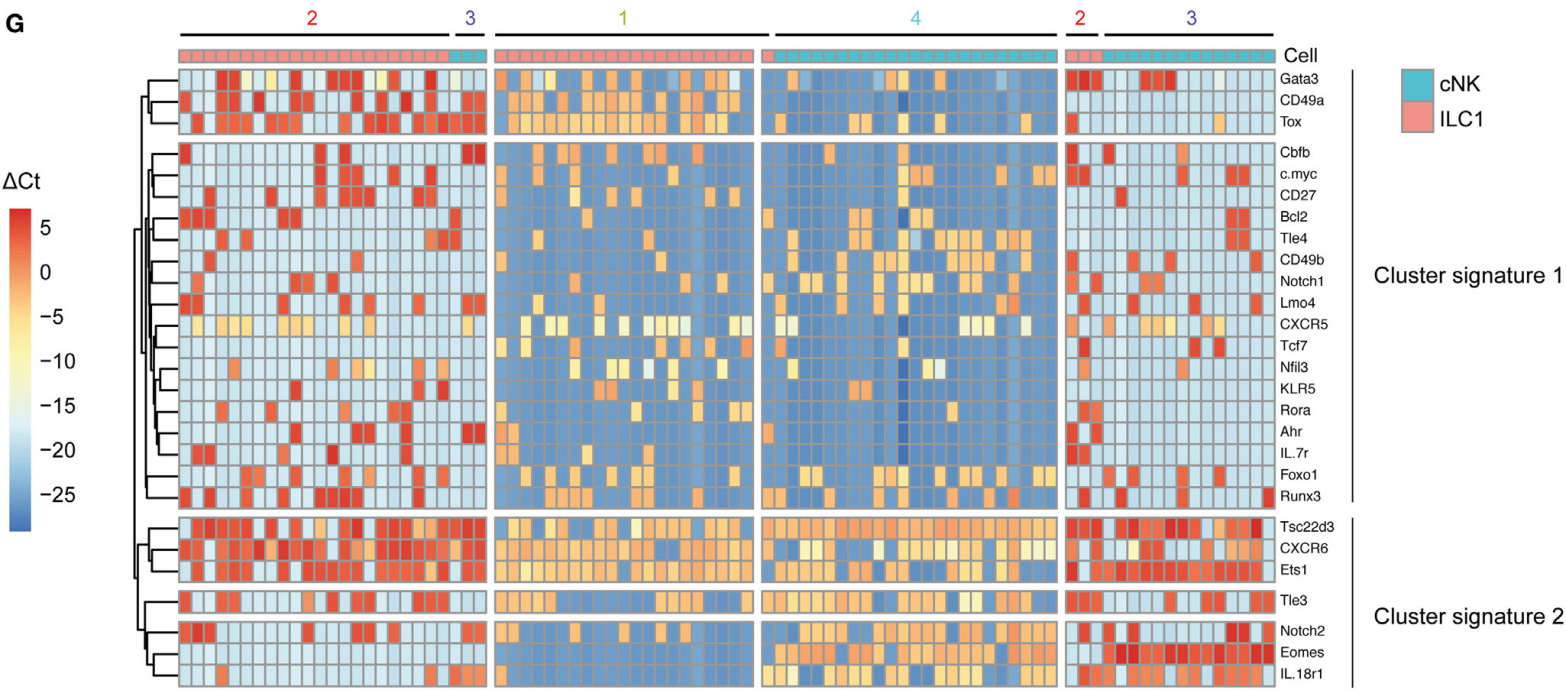
Group 1 innate lymphoid cell is composed of four distinct populations in the liver at homeostasis. **(A)** Single cells were sorted for transcriptomic analyses using the Biomark technology. Hepatic lineage negative CD45-positive cells (lin^−^ CD45^+^) were separated into type 1 helper innate lymphoid cells (ILC1) (CD4^−^ NKp46^+^ NK1.1^+^ CD49a^+^ CD49b^−^) and conventional NK (cNK) cells (CD4^−^ NKp46^+^ NK1.1^+^ CD49a^−^ CD49b^+^). **(B)** Application of t-SNE to single cells using 48 genes expression separates 48 ILC1 and 48 cNK into two distinct subsets. **(C)** Phenograph algorithm clustering performed and plotted on t-SNE graph, showing four different clusters among hepatic ILC1 and cNK. **(D)** Density plot of *Eomes* gene expression show four distinct types of expression among phenograph clusters of hepatic ILC1 and cNK. **(E)** The variance mean of delta Ct across expressed genes allows the selection of genes that are differentially expressed (variance > 60) with a significant expression (dCt from −20.5 to 0). **(F)** Frequency of hepatic ILC1 and cNK expressing Notch1 and/or Notch2. **(G)** Heatmap with unsupervised hierarchical clusters of gene selected in panel **(D)**.

Mutually exclusive expression of CD49a and CD49b separates the NKp46^+^ NK1.1^+^ population into ILC1 and cNK cells in the murine liver (Figure 1A). Purified single ILC1 and cNK cells were subjected to multiplex transcriptional analysis, as described (18). We analyzed 48 transcripts known or supposed to be expressed in type 1 ILC with some of them also being possible Notch pathway targets. A t-SNE analysis of the data set indicated that both ILC1 and cNK cells were subdivided into two populations (Figure 1B), populations 1 and 2 for ILC1 and populations 3 and 4 for cNK (Figure 1C). Surprisingly, population 1 of ILC1 clustered closer to the cNK population 4 rather than to its ILC1 counterpart. Inversely, population 3 of cNK clustered closer to ILC1 population 2 (Figure 1C). We ascertained that cNK express high levels of Eomes transcripts and that ILC1 subsets had low expression levels (Figure 1D). Unexpectedly, Eomes gene expression is found as highly variable and different levels are observed for these four populations (Figure 1E). We then restricted our analysis to the genes that were the most variable (>60 of variance) and significantly expressed (mean values selection between 0 and −20) (Figure 1E). With *Eomes, Gata3, Tox, Notch2, Runx3*, and *Itga1* were among the most variable transcripts (Figure 1E). Notch receptors were expressed more frequently in cNK cells than in ILC1 (Figure 1F). Notch2^+^ cells were more frequent than Notch1^+^ cells (Figure 1F) and cells expressing both Notch1 and Notch2 transcripts represented 9% of ILC1 and 30% of cNK. It is interesting to notice that population 1 of Eomes^lo^ ILC1 was enriched in Notch expressing cells compared with Eomes^−^ ILC1 (population 2). An unsupervised hierarchical cluster was constructed based on this restricted list of genes. The segregation of ILC1 and cNK into four different subsets was consistent with the t-SNE analysis (Figure 1G). The genes could be separated into two cluster signatures. The first comprises genes directly related to the Notch pathway (*Notch1, Gata3, Ahr, Tcf7, IL7ra*, and *c-myc*) and genes that define the identity of ILC1 versus cNK (CD49a, *Cd27*, and *Il7ra*). In the second cluster, *Notch2* together with *Cxcr6, Eomes*, CD49b, and *Il18r1* define the signature 2 and also cNK identity. Correlation heatmaps confirmed that most of genes among each signature are correlated (Figure S1A in Supplementary Material). A good correlation is shown between most genes of signature 1 with a strong correlated core for Tcf7, CD27, Rora, Klr5, Ahr, and Il7r (Figures S1A,B in Supplementary Material). A good correlation is described between Eomes, IL18r1, Tsc22d3, CD49b, and Tle4 for the signature 2 (Figure S1C in Supplementary Material). The data suggest that the Notch signaling pathway could play a role in the specification of the subsets of ILC1 and cNK in the liver.

### RBPJ-Deficient Type 1 ILC Have Different Characteristics

**Figure 2:**
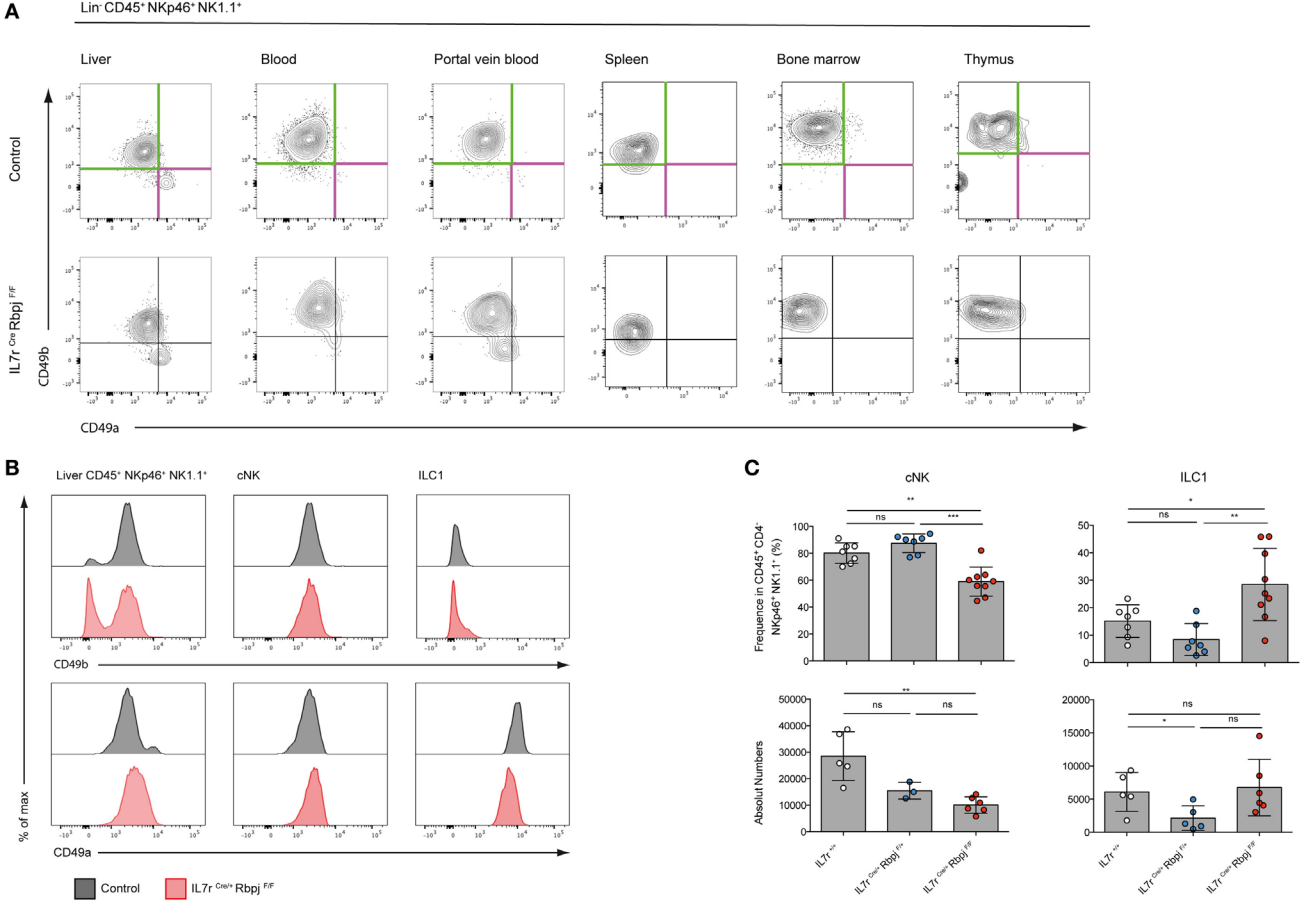

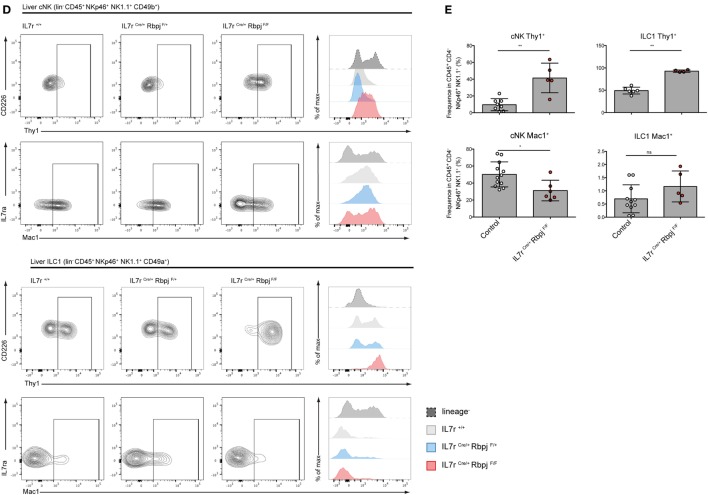
Altered phenotype of NKp46^+^ NK1.1^+^ cells in IL7r^Cre^ Rbpj^F/F^ mice. **(A)** Expression of CD49a (purple) and CD49b (green) in lin^−^ CD45^+^ NKp46^+^ NK1.1^+^ cells from the liver, blood, portal vein blood, spleen, bone marrow, and thymus of control IL7r^Cre^ Rbpj^F/+^ mice (top panel) and IL7r^Cre^ Rbpj^F/F^ mice (bottom panel). **(B)** Surface expression of CD49b (top panel) and CD49a (bottom panel) on total liver lin^−^ CD45^+^ NKp46^+^ NK1.1^+^ cells, hepatic conventional NK (cNK) and type 1 helper innate lymphoid cells (ILC1) in control IL7r^Cre^ Rbpj^F/+^ mice (gray) and IL7r^Cre^ Rbpj^F/F^ (red). **(C)** Frequency and absolute numbers of hepatic cNK and ILC1 in IL7r^+/+^ (white), IL7r^Cre^ Rbpj^F/+^ (blue), and IL7r^Cre^ Rbpj^F/F^ (red) mice. **(D)** Surface expression of Thy1 and Mac1 on hepatic cNK (top panel) and ILC1 (bottom panel) in IL7r^+/+^ (gray), IL7r^Cre^ Rbpj^F/+^ (blue), and IL7r^Cre^ Rbpj^F/F^ (red) mice. Lineage-negative cells were used as control for histograms (dashed gray). **(E)** Frequency of hepatic cNK and ILC1 expression of Thy1 (top panel) and Mac1 (bottom panel) in control IL7r^Cre^ Rbpj^F/+^ mice (white) and IL7r^Cre^ Rbpj^F/F^ (red).

We analyzed type 1 ILC (Lin^−^ CD45^+^ NK1.1^+^ NKp46^+^ cells) in Il7r^Cre^ Rbpj^F/F^ mice to define the role of the canonical Notch signaling pathway in the maturation of this population (11). CD49a and CD49b expression that distinguishes hepatic ILC1 from cNK cells was tested in the spleen, BM, and thymus of Notch-competent and Notch-deficient mice (Figure 2A). In Notch-deficient mice, CD49a levels were decreased in most ILC1 while CD49b levels remained unchanged. Interestingly, a CD49a^lo^ CD49b^−^ population appears in the circulation especially in the PVB (Figure 2A). Notch-deficient hepatic ILC1 showed decreased levels of CD49a (Figure 2B) and increased frequencies and absolute numbers (Figure 2C).

We then analyzed mice where the Notch signaling pathway was defective in hematopoietic cells (Vav^Cre^ Rbpj^F/F^) or downstream of Notch2 (IL7r^Cre^ Notch2^F/F^) (Figure S2A in Supplementary Material). In Notch2-deficient liver ILC (IL7r^Cre^ Notch2^F/F^), CD49a and CD49b expression on type 1 ILC was unchanged contrasting with the decreased levels of CD49a in RBPJ-deficient ILC irrespective of whether deletion of RBPJ occurred in IL7r or in Vav-expressing cells (Figure S2B in Supplementary Material). Interestingly, while RBPJ deletion resulted in an increased frequency of ILC1, the Notch2 deletion induced an opposite effect with a decreased frequency of ILC1 suggesting non-redundant roles of Notch1 and Notch2 (Figure S2C in Supplementary Material) that was not due to differences in proliferation (Figure S3 in Supplementary Material). The analyses of Thy1 expression showed an increase from 10 to 50% in Notch-deficient cNK and to virtually 100% in Notch-deficient ILC1. Mac-1 expression among cNK showed a significant decrease (Figures 2D,E).

**Figure 3:**
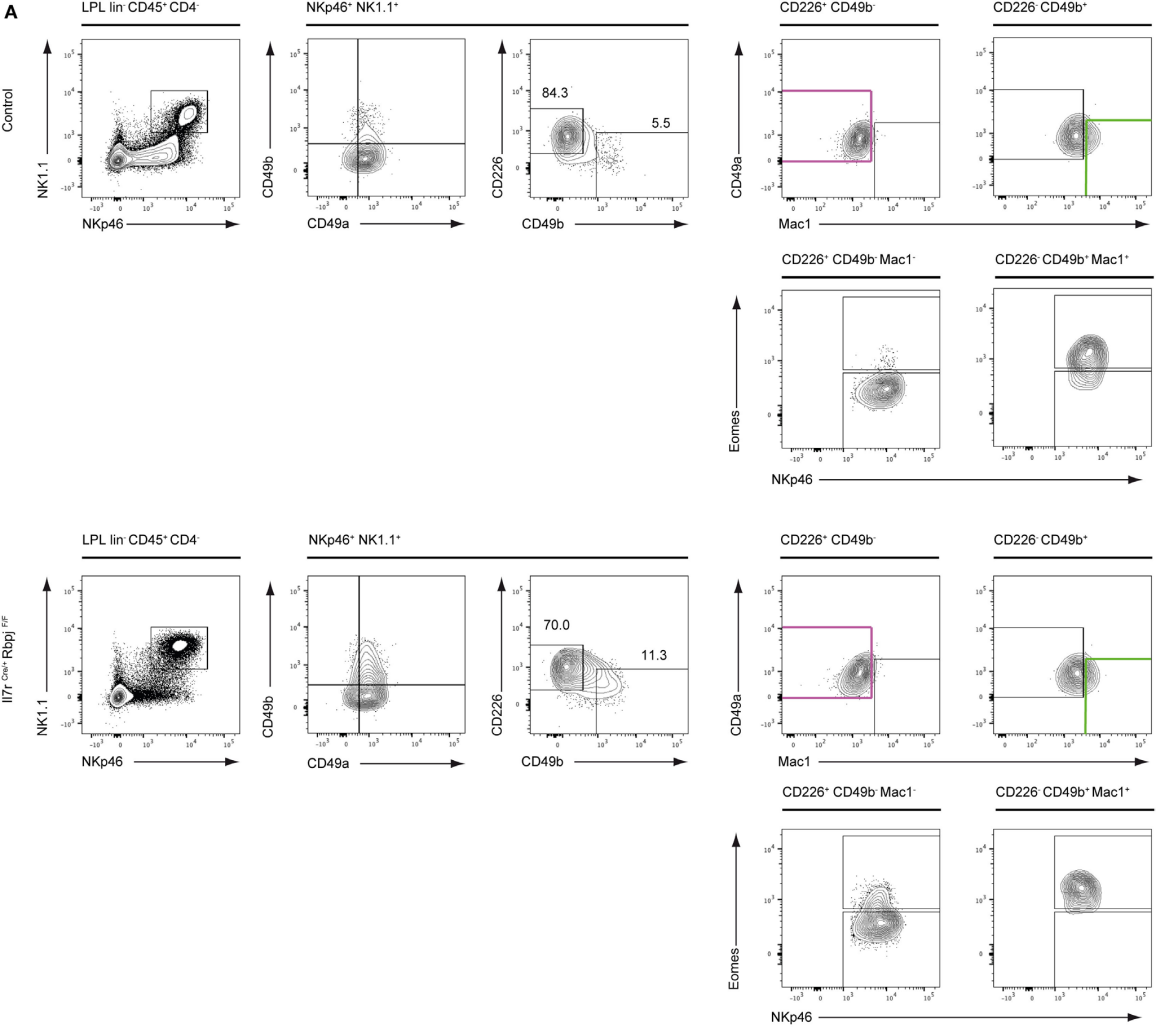

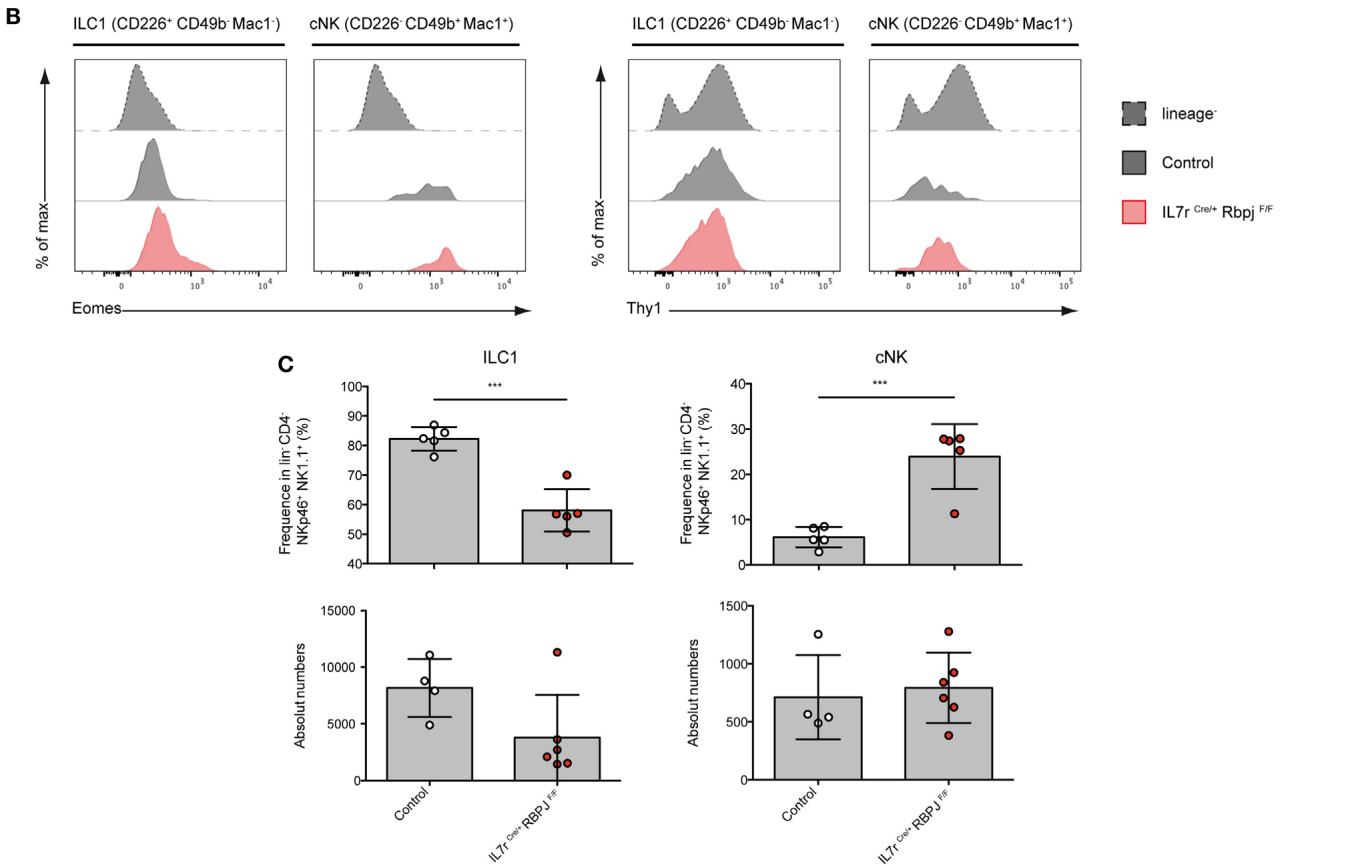
Conventional NK (cNK) cells are increased in the lamina propria lymphocytes (LPL) of IL7r^Cre^ Rbpj^F/F^ mice. **(A)** Flow cytometry of control IL7r^Cre^ Rbpj^F/+^ mice (top panel) and IL7r^Cre^ Rbpj^F/F^ LPL (bottom panel). Repartition of type 1 helper innate lymphoid cells (ILC1) (lin^−^ CD45^+^ CD4^−^ NKp46^+^ NK1.1^+^ CD226^+^ CD49b^−^ CD49a^+^ Mac1^−^, in purple) and NK (lin^−^ CD45^+^ CD4^−^ NKp46^+^ NK1.1^+^ CD49b^+^ CD226^−^ CD49a^−/+^ Mac1^+^, in green) in lamina propria (LP). **(B)** Eomesodermin (Eomes) and Thy1 expression in LP ILC1 and cNK in control IL7r^Cre^ Rbpj^F/+^ mice (gray) and IL7r^Cre^ Rbpj^F/F^ (red). Lineage-negative cells were used as control for expression (dashed gray). **(C)** Frequency and absolute numbers of LP ILC1 and cNK in control IL7r^Cre^ Rbpj^F/+^ mice (white) and IL7r^Cre^ Rbpj^F/F^ (red).

We have previously shown that type 1 ILC (Lin^−^ NKp46^+^ NK1.1^+^ cells) in the intestinal lamina propria (LP) were not affected by RBPJ deletion (17). However, the changes in the expression levels of CD49a and CD49b described above led us to reevaluate the representation of the subsets of Notch-deficient type 1 ILC1 in the LP. In LP, CD49a and CD49b could not strictly separate ILC1 from cNK cells as numerous cells express both markers (Figure 3A). We designed a panel of surface markers that allowed the enrichment of NKp46^+^ NK1.1^+^ cells into Eomes^−^ versus Eomes^+^ subsets. Cells separated as CD226^+^ CD49b^−^ Mac1^−^ are enriched for Eomes^−^ ILC1 and CD226^−^ CD49b^+^ Mac1^+^ cells are enriched into Eomes^+^ cNK cells (Figure 3A). We found that Notch-deficient CD226^+^ CD49b^−^ Mac1^−^ ILC1 comprises 15% of Eomes^+^ cells whereas the enriched cNK subset is exclusively composed of Eomes^+^ cells (Figures 3A,B). Similar to liver type 1 ILC all subsets in the LP have increased Thy1 levels (Figure 3B). Consistent with our previous observations, the absolute numbers of ILC1/cNK remained unchanged in RBPJ-deficient compared with control mice (Figure 3C). Altogether, these results indicated that the Notch signaling pathway was modulating several properties of the type 1 ILC including expression of transcription factors, integrins, and the capacity to circulate.

### Type 1 ILC Functions Are Altered in RBPJ-Deficient Cells

**Figure 4:**
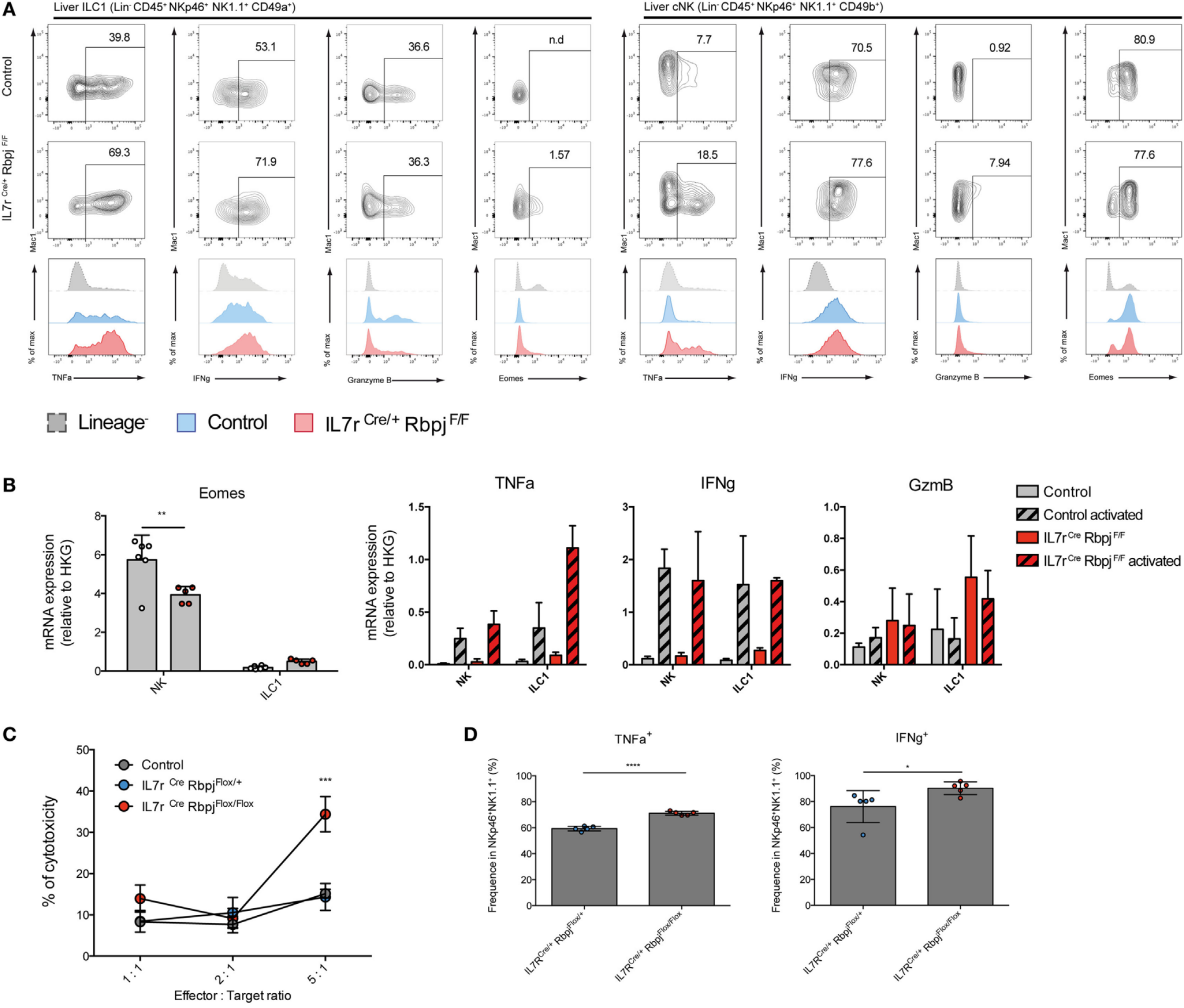

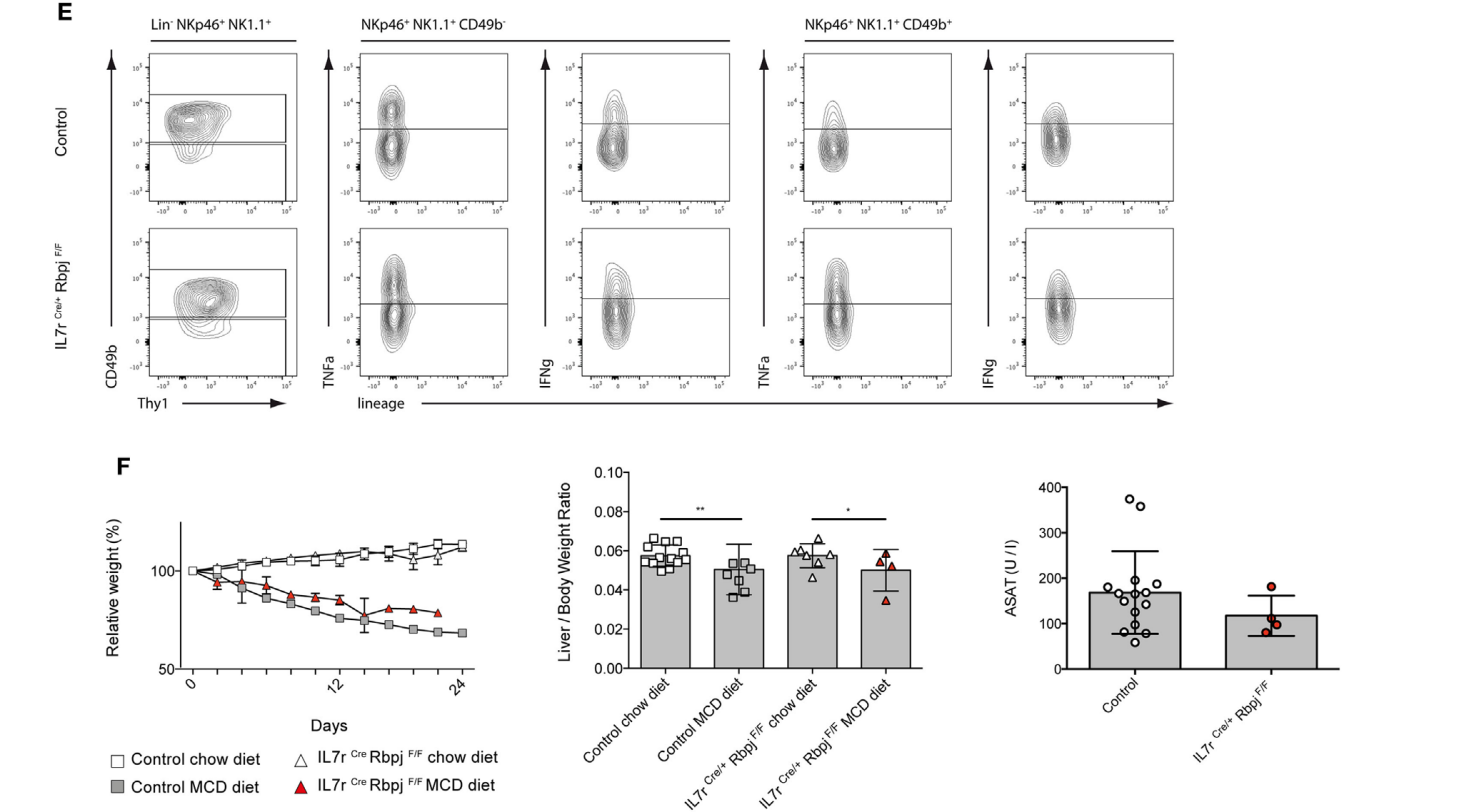
Altered cytokine profile of NKp46^+^ NK1.1^+^ cells in IL7r^Cre^ Rbpj^F/F^ mice. **(A)** Expression of TNFa, IFNg, granzyme B, and eomesodermin (Eomes) in hepatic type 1 helper innate lymphoid cells (ILC1) (left panel) and conventional NK (cNK) (right panel) of control IL7r^Cre^ Rbpj^F/+^ mice (top panel) and IL7r^Cre^ Rbpj^F/F^ mice (middle panel). Levels of expression were compared (bottom panel) between control IL7r^Cre^ Rbpj^F/+^ mice (blue) and IL7r^Cre^ Rbpj^F/F^ mice (red). Lineage-negative cells were used as control for surface expression (dashed gray). **(B)** Expression of *Eomes, Tnfa, Ifng*, and *Gzmb* mRNA in control IL7r^Cre^ Rbpj^F/+^ mice (gray) and IL7r^Cre^ Rbpj^F/F^ mice (red). For *Tnfa, Ifng*, and *Gzmb*, mRNA expression was also tested on activated cells (dashed histograms). **(C)** Splenic cNK cells killing capacity in control C57BL/6J (gray), IL7r^Cre^ Rbpj^F/+^ (blue), and IL7r^Cre^ Rbpj^F/F^ (red) mice. **(D)** Frequency of cNK expressing TNFa and IFNg during cytotoxicity assay. **(E)** Cytokine profile of hepatic ILC1 and NK cells in control IL7r^Cre^ Rbpj^F/+^ (top panel) and IL7r^Cre^ Rbpj^F/F^ mice (bottom panel) after methionine-choline deficient (MCD) diet. **(F)** Relative weight and liver/body weight ratio in control IL7r^Cre^ Rbpj^F/+^, IL7r^+/+^ Rbpj^F/+^, and IL7r^+/+^ Rbpj^F/+^ mice (square) after chow (white) and MCD diet (gray) and in IL7r^Cre^ Rbpj^F/F^ mice (triangles) after chow (white) and MCD diet (red). Aspartate aminotransferase (ASAT) levels of control IL7r^Cre^ Rbpj^F/+^, IL7r^+/+^ Rbpj^F/+^, and IL7r^+/+^ Rbpj^F/+^ (white) and IL7r^Cre^ Rbpj^F/F^ mice (red) after MCD diet.

We then assessed the functional properties of RBPJ-deficient hepatic ILC1 and cNK cells. We found that, after activation with PMA-ionomycin, TNFa and IFNg secretion by RBPJ-deficient ILC1 and cNK cells were more strongly increased compared with control cells (Figure 4A). TNFa was also more expressed by ILC1 than cNK, whereas IFNg was produced by most hepatic ILC1 and cNK, after Notch depletion. GzmB production by ILC1 remained unchanged while it was produced by few cNK Mac1^+^, in absence of the Notch signaling pathway (Figure 4A). Similar to those from LP, hepatic RBPJ-deficient ILC1 comprise a fraction of Eomes^+^ cells, whereas a subset of Eomes^−^ Mac1^−^CD49b^+^ cells becomes more prominent among cNK cells. These differences in Eomes expression were also apparent in qRT-PCR (Figure 4B). Overall, the mRNA expression for *Tnfa, Gzmb*, and *Ifng* genes confirmed the increase of protein levels and high production of IFNg (Figure 4B). Similar experiments done in hepatic ILr7^Cre^ Notch2^F/F^ mice showed consistent increase of TNFa and IFNg production by Notch 2-deficient type 1 ILC (Figure S4 in Supplementary Material). RBPJ-deficient splenic NKp46^+^NK1.1^+^ cells showed significantly increased lytic abilities on YAC-1 mouse lymphoma cells (Figure 4C) that correlated with an increase frequency of cells capable to produce TNFa and IFNg (Figure 4D). To assess the *in vivo* functions of Notch-deficient type 1 ILC in inflammatory conditions, we used a model of liver damage with inflammation, immune infiltration, and fibrosis (19) induced by methionine-choline deficient (MCD) diet. Under MCD diet, RBPJ-deficient mice showed no differences in frequency of TNFa^+^ and IFNg^+^ cells (Figure 4E), in weight loss, and in the ratio of liver size versus body weight (Figure 4F). The levels of the circulating transaminase aspartate aminotransferase were also not different after 24 days of MCD diet (Figure 4F).

Taken together, these experiments indicated that RBPJ-deficient type 1 ILC had increased levels of inflammatory cytokines and increased cytotoxic activity that are not modified by a liver inflammatory inducing diet.

### ILC1 and cNK Have Variations in Gene Expression After Abrogation of the Notch Signaling Pathway

**Figure 5:**
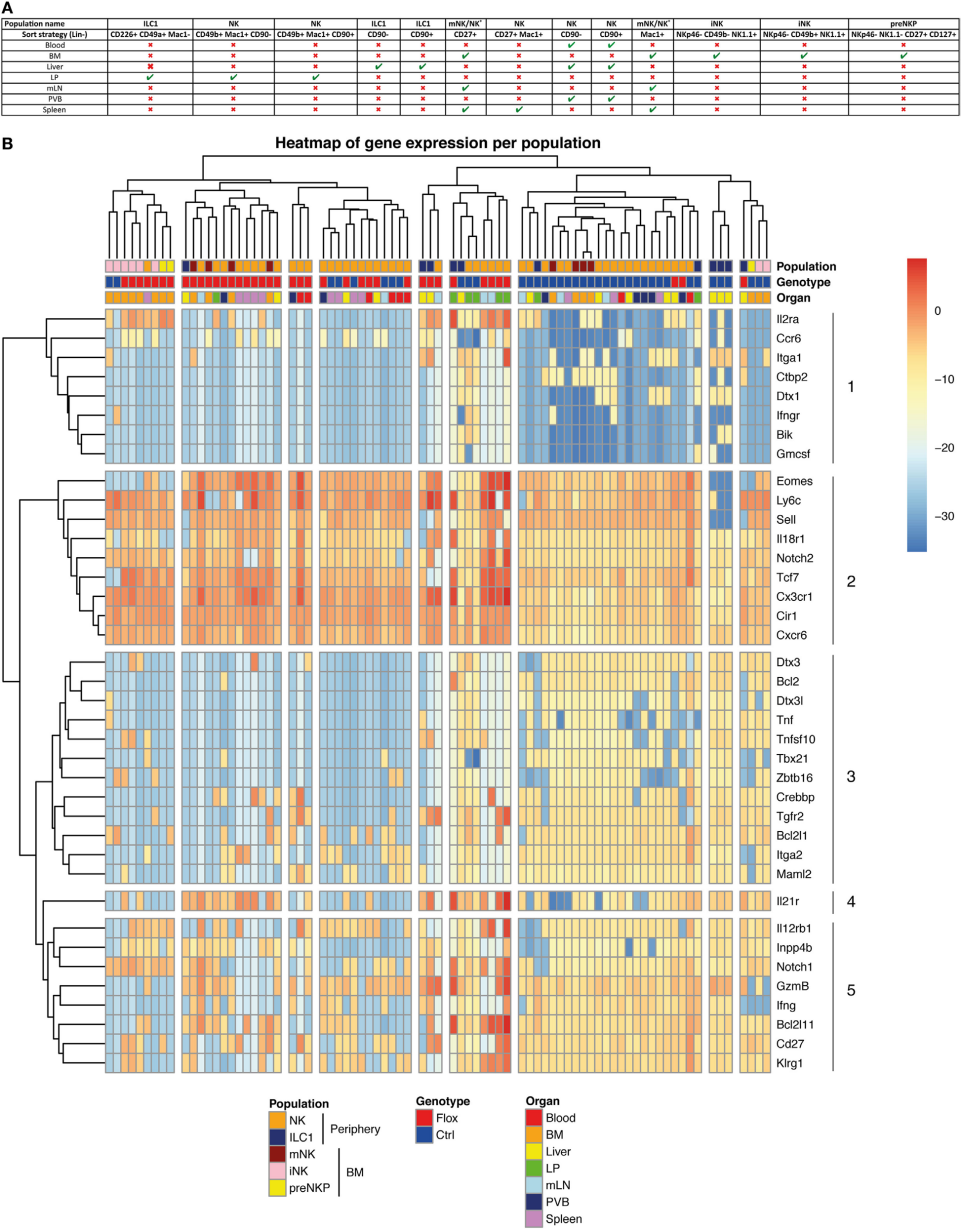
Molecular signature heterogeneity of group 1 innate lymphoid cell (ILC) in IL7r^Cre^ Rbpj^F/+^ and IL7r^Cre^ Rbpj^F/F^ mice. **(A)** Sorting strategy of group 1 ILC cells in the different tissues **(B)** Heatmap of genes expression in group 1 ILC of IL7r^Cre^ Rbpj^F/+^ and IL7r^Cre^ Rbpj^F/F^ mice in blood, bone marrow (BM), liver, lamina propria (LP), mesenteric lymph nodes (mLN), portal vein blood (PVB), and spleen. Cluster of cells and genes were obtained using hierarchical clustering.

To understand the role of Notch signaling pathway in ILC1 and cNK cells, we performed multiplex quantitative transcriptional analysis of 41 immune genes in these different populations from various organs. We used small numbers of cells (25 cells per subset) to reduce the averaging generated by population-level studies. We sorted the type 1 ILC populations of Notch-competent (Ctrl) and -deficient (Flox) mice in distinct organs according to the strategy outlined in Figure 5A.

We only analyzed samples expressing all three “housekeeping” genes and did an unsupervised hierarchical clustering analysis of the transcriptional profiles from 79 samples (Figure 5B). The population identity is indicated in the first line with a color code for cNK, ILC1 versus BM subsets of preNKP, iNK, and mNK cells. The genotype of the subset analyzed is indicated in the second line with a color code for Notch-competent (Ctrl-Blue) and Notch-deficient (Flox-Red) subsets and the third line indicates the tissue of origin.

The separation between the RBPJ-competent and -deficient samples was evident in most samples. Only, some splenic samples, exclusively composed of mature cNK cells, showed similar distribution of RBPJ-competent and -deficient subsets and clustered together with RBPJ-competent blood and RBPJ-deficient liver cNK samples. This indicates that RBPJ-deficient cNK cells from liver resemble cNK splenic subsets, insensitive to Notch inactivation. It also suggests that the canonical Notch pathway maintains an identity of cNK cells in liver, LP, mesenteric lymph node (mLN), and PVB different from that found in the spleen and circulation.

Two subsets of BM iNK clustered with their Notch-deficient counterparts. Because all type 1 ILC and their precursors express *Tcf7* and these two samples express low levels of *Tcf7* and *Cd27*, we concluded that they contained few NK precursors. We decided not to eliminate them from the analysis, as they did not alter its global architecture of the hierarchy.

As discussed above, while surface expression of CD49a and CD49b separated hepatic ILC1 from cNK their separation by transcriptional profiling is less stringent. Indeed, even if most ILC1 subsets clustered together and separated from cNK, a few samples of hepatic ILC1 are interspersed with cNK cells. Notch-competent hepatic ILC1 subsets clustered with intestinal ILC1 and BM precursors (preNKp + iNK), whereas Notch-deficient hepatic ILC1 cluster together and were in the vicinity of mLN and intestinal LP cNK populations.

Our analysis allowed the separation of genes that varied with the activity of the Notch pathway. Genes in cluster 2 were upregulated and conversely genes in cluster 3 were dowregulated, in most Notch-deficient samples. We observe that interfering with the canonical Notch pathway in cNK cells of the enterohepatic axis led to the upregulation of *Eomes, Ly6c, Sell, Il18r1, Notch2, Tcf7, Cx3cr1, Cir1*, and *Cxcr6*. Nearly all were also upregulated in ILC1 subsets with the exception for *Sell* that is never found in this population. Despite being generally increased, *IL18r1* and *Tcf7* were undetectable in few ILC1 Notch-deficient samples illustrating a variable expression in this subset.

A group of genes related to ILC1 signature (*Il21r, Lnpp4b*, and *Tnfsf10*) were also upregulated in most Notch-deficient ILC1. *Itga1* was maintained in most hepatic RBPJ-deficient ILC1, although the BM and LPL cNK subsets silenced *Itga1* after RBPJ depletion. *Tnfsf10* expression was also downregulated after RBPJ depletion in cNK subsets. Other genes such as *IL12rb1, IL2ra*, and *Notch1* displayed increased expression after Notch depletion.

With the exception of *Tcf7* that was upregulated, most other known direct targets of the canonical Notch pathway comprising *Dtx1, Dtx3, Dtx3l, Zbtb16, Bcl2, Bik*, and *Tbx21* were silenced in Notch-deficient cells.

Genes implicated in apoptosis (*Bcl2l1, Crebbp*, and *Bcl2*) were down-modulated, whereas *Bcl2l11* showed increased expression in Notch-deficient cells.

*Maml2*, a co-activator of the Notch pathway, is decreased, whereas *Cir* a RBPJ co-repressor is upregulated, in RBPJ-deficient cells. Splenic and circulating cNK subsets that were scattered within Notch-competent and -deficient genotypes did not express *Dtx1, Dtx3, Dtx3l, Crebbp, Bcl2, Zbtb16*, and *Tbx21*, thus appearing Notch independent. These subsets also did not express *Il21r, Tnf, Tnfsf10, Lnpp4b*, and *Tgfr2*.

To better visualize the genes that are co-modified by inactivation of the Notch pathway, we built a heatmap (Figure S5 in Supplementary Material) that clusters together genes that vary in a similar manner comparing RBPJ-proficient and -deficient cells. Gene enrichment analysis of the clusters thus obtained allowed identifying some hallmark for different pathways. *Il2ra, Eomes, IL18r1, Gzmb*, and *Sell* that are co-regulated belong to the inflammatory response genes and to those that are stimulated by Stat5 in response to IL2 stimulation. It is interesting to notice that these genes are related to *Notch2, Tcf7, Ly6c*, and the chemokine receptors *Cx3cr1* and *Cxcr6*.

A few genes at the bottom of the heatmap (*Tnfsf10, Bcl2*, and *Bcl2l1*) (Figure S5 in Supplementary Material) are also associated to the Stat5/IL2 pathway. Moreover, they correlated with genes implicated in the Notch signaling (*Notch1, Maml2, Crebbp, Dtx3*, and *Dtx3l*) and with Notch target genes (*Tbx21, Zbtb16*). *Itga2* correlated with *Notch1, Dtx3, Dtx3l*, and *Tbx21*, whereas *Itga1* correlated to *Dtx1, IL21r*, and *Ctbp2*.

We have previously observed after stimulation of RBPJ-deficient ILC1 subsets, a marked upregulation of *Tnfa* transcripts with similar levels for *Infa* and *Gzmb* (Figure 4B). However, in homeostatic conditions, *Tnfa, Ifng*, and *Gm-csf* transcripts were decreased in opposition to an increase for those coding *Gzmb*. Finally, we found that genes differentially regulated by the Notch pathway in type 1 ILC had a highly conserved binding site motif for NFAT and FOXO4. This suggests interactions between NFAT/FOXO4 and the Notch signaling pathway in regulating immune processes in these cells.

### RBPJ Deficiency Increases Type 1 ILC Control at the Initial Stages of Hepatic Tumor Development

**Figure 6:**
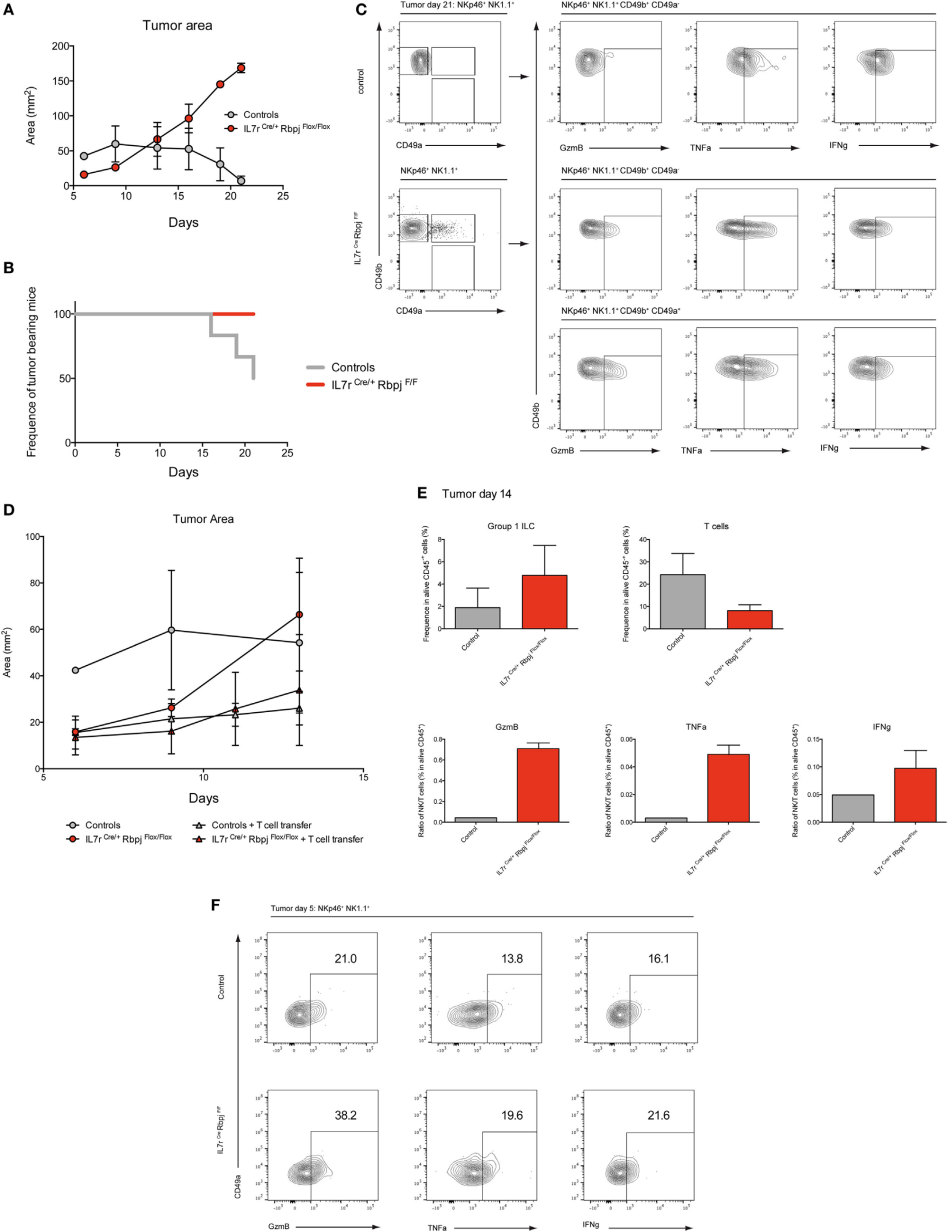
Notch signaling participates to early antitumoral activity. **(A)** Tumor area after subcutaneous injection of 3 × 10^6^ Hepa1.6 cells in control IL7r^Cre^ Rbpj^F/+^, IL7r^+/+^ Rbpj^F/+^, and IL7r^+/+^ Rbpj^F/F^ (gray) and IL7r^Cre^ Rbpj^F/F^ mice (red). **(B)** Frequency of mice with a tumor after subcutaneous injection of 3 × 10^6^ Hepa1.6 cells in control IL7r^Cre^ Rbpj^F/+^, IL7r^+/+^ Rbpj^F/+^, and IL7r^+/+^ Rbpj^F/F^ (gray) and IL7r^Cre^ Rbpj^F/F^ mice (red). 50% of control mice rejected the tumor after 21 days. **(C)** Group 1 innate lymphoid cell (ILC) tumor infiltrates in control IL7r^Cre^ Rbpj^F/+^, IL7r^+/+^ Rbpj^F/+^, and IL7r^+/+^ Rbpj^F/F^ mice (top panel) and IL7r^Cre^ Rbpj^F/F^ mice (bottom panel) 21 days after Hepa1.6 cells injection. **(D)** Tumor area after subcutaneous injection of 3 × 10^6^ Hepa1.6 cells in control IL7r^Cre^ Rbpj^F/+^, IL7r^+/+^ Rbpj^F/+^, and IL7r^+/+^ Rbpj^F/F^ (gray dot), IL7r^Cre^ Rbpj^F/F^ mice (red dot), control IL7r^Cre^ Rbpj^F/+^, IL7r^+/+^ Rbpj^F/+^, and IL7r^+/+^ Rbpj^F/F^ mice with T cell transfer (gray triangle) and IL7r^Cre^ Rbpj^F/F^ mice with T cell transfer (red triangle). **(E)** Frequency of group 1 ILC and CD3-positive cells in tumor of control IL7r^Cre^ Rbpj^F/+^, IL7r^+/+^ Rbpj^F/+^, and IL7r^+/+^ Rbpj^F/F^ (gray), IL7r^Cre^ Rbpj^F/F^ mice (red) with T cell transfer 14 days after Hepa1.6 cells injection. Ratio of group 1 ILC on T cell expressing granzyme B (GzmB), TNFa, and IFNg. **(F)** Expression of GzmB, TNFa, and IFNg in group 1 ILC tumor infiltrates of IL7r^Cre^ Rbpj^F/+^, IL7r^+/+^ Rbpj^F/+^, and IL7r^+/+^ Rbpj^F/F^ control (top panel) and IL7r^Cre^ Rbpj^F/F^ mice (bottom panel) 5 days after Hepa1.6 cells injection.

To assess the effect of RBPJ deficiency on hepatic type 1 ILC function, we chose a model of hepatocellular carcinoma by injection a Hepa1–6 mouse liver cancer cell line. It was shown that cNK and T cells are important in the control of the tumors *via* IFNg and lytic granules (20). Three weeks after subcutaneous injection of Hepa1–6 cells, the area of the tumor was significantly increased in RBPJ-deficient mice compared with control mice (Figure 6A). Moreover, at 21 days post-transplantation, RBPJ-deficient mice showed no sign of tumor rejection while 50% of control mice were tumor free (Figure 6B). The analysis of the tumor-infiltrating cells indicated that CD49a^+^ CD49b^+^ cNK subset was only found in RBPJ-deficient animals (Figure 6C) that were more efficient than the conventional cNK subset in secreting GzmB and TNFa (Figure 6C). However, since RBPJ-deficient animals contain defective T cell subsets, they are not able to efficiently eliminate the tumors despite more cytotoxic cNK populations. Therefore, the tumor area difference between RBPJ-competent and -deficient mice starts around day 14 post-injection (Figure 6A). Consistent with a role of T cells in tumor rejection, we found infiltrated CD4^+^ and CD8^+^ T cells 14 days after injection in C57Bl6 mice (Figure S6 in Supplementary Material). T cells transferred resulted in the control of RBPJ-deficient mice tumor growth similar to that in Notch-competent mice (Figures 6D,E). Tumor-infiltrating cells were analyzed revealing a lower frequency of T cells and conversely a higher frequency of type 1 ILC in RBPJ-deficient mice compared with controls (Figure 6E). We showed that even if T cells secreting GzmB, TNFa, and IFNg were more numerous the cytotoxicity produced by cNK populations against the tumor was higher in RBPJ-deficient mice, even after T cell transfer (Figure 6E). Because RBPJ-deficient controlled better than RBPJ-competent mice the expansion of the tumor at early time points (Figure 6A), we analyzed the type 1 ILC composition and functions 5 days after tumor injection, a time point at which no infiltrating T cells could be detected. In RBPJ-deficient mice, the CD49a^+^ subset was still absent but the intratumoral cNK cells were more prone to release GzmB, TNFa, and IFNg (Figure 6F). We propose that the canonical Notch signaling pathway is involved in the downregulation of cytotoxic capacities of specific cNK cell subsets and also in the control of CD49a expression levels on recruited type 1 ILC populations.

## Discussion

In the liver, cNK and ILC1 are more heterogeneous than initially thought. Contrary to CD49b^+^ cNK, resident hepatic ILC1 have been defined as Eomes^−^ CD49a^+^ CD49b^−^ T-bet dependent (2, 21–24). We distinguished here two subsets of ILC1 and of cNK based on the expression of a limited set of transcripts. Surprisingly, Eomes transcripts were found expressed in one of the hepatic ILC1 subgroup and they were differentially expressed among cNK subsets. Differences in tissue ILC1 populations have been attributed to the environment, particularly in glands where ILC1 express both Eomes and T-bet (25). This made us consider the possibility that Eomes levels may be actively suppressed in hepatic ILC1. Since Eomes expression is repressed in T-bet^+^ hepatic ILC1 (2), and that T-bet is a possible target of the Notch pathway, we investigated whether the Notch pathway acts on hepatic type 1 ILC.

Using single-cell transcriptomic analyses, we confirmed that nearly half of hepatic ILC1 and cNK cells expressed Notch receptors. We found that expression is heterogeneous as cells could either be Notch1^+^, Notch2^+^, or both. Hence, we suspected that the Notch signaling pathway could also play a role on different characteristics of type 1 ILC from other organs. Therefore, by selecting genes belonging to type 1 ILC developmental program and to the Notch signaling pathway, we designed a comparative transcriptomic study on numerous small populations of type 1 ILC subsets from diverse tissues. We showed that Notch pathway is actively operating in populations from most tissues, except for spleen where a substantial amount of cells was found to be Notch insensitive.

We found that RBPJ deficiency was associated with the reduction of the *Tbx21* gene expression in half hepatic ILC1 validating our hypothesis of the Notch pathway implication on hepatic subset specification and functions. We assume that T-bet might also be activated by other signaling pathways because the other half of ILC1 that are not expressing Notch receptors should have a path to upregulate T-bet. We observed identical changes of ILC1 and cNK transcriptional program in other organs. In a previous study, we reported that T-bet levels were maintained in type ILC1 in the intestinal LP (17). However, in our previous study, the Notch sensitive population was diluted among Notch insensitive cells masking the effect of the Notch signaling depletion. Therefore, to overcome this limitation, we used only 25 cells per population in this comparative transcriptomic assay. In the absence of the Notch signaling pathway, we also detected an increase of Eomes expression in ILC1 and cNK subsets both at transcriptional and protein levels. The presence of a new immature Eomes^−^ cNK population in the liver diluted the Eomes transcript levels from the global population as observed in Figure 4. The expression of Eomes is probably due to the decrease of T-bet, as previously observed in T-bet-deficient cNK cells (2, 26). Moreover, T-bet^−^ cNK cells fail to express Mac1 and maintain their CD27 levels (27, 28). Consistently, we observed a decrease of Mac1 expression in liver cNK cells and the presence of CD27^+^ Mac1^−^ immature subset. The presence of these immature subsets might be linked to the decrease of T-bet expression in type 1 ILC. It has been hypothesized that hepatic ILC1 depends on T-bet for their development, although no direct evidence has been provided. In our RBPJ-deficient model, it is possible that all Notch-dependent ILC1 subsets are absent because they are not able to differentiate *in situ*. This immature subset could therefore represent accumulating ILC1 precursors that could not progress to the T-bet^+^ stage. On other hand, peripheral immature subsets could also represent accumulating immature cNK cells. It has been shown that environment could induce conversion between ILC1 and cNK (25, 29, 30). Our study illustrates a probable hepatic ILC1 differentiation into cNK where the absence of the Notch pathway leads to the induction of Eomes *via* the downregulation of its regulator T-bet. Consistent with this hypothesis, in T-bet-deficient mice, precursors in the BM show an increase of the immature CD27^+^ and a decrease of the mature Mac1^+^ populations (Figure S7 in Supplementary Material). Clustering of BM RBPJ-competent and -deficient subsets confirmed a role for Notch signaling in the early development of type 1 ILC. Even if T-bet levels are actively maintained low in BM cNK precursors, T-bet is expressed at the immature CD27^+^ Mac1^−^ stage. T-bet has been shown to control S1P5 expression which participates to cNK trafficking (31, 32). As in T-bet-deficient mice, we also found more cNK cells in the BM (Figure S7 in Supplementary Material) of RBPJ-deficient mice while their ratio were decreased to the expense of ILC1 in the periphery (32, 33). We proved that the increase of ILC1 was not due to excessive proliferation and propose that the Notch induced decrease of T-bet results in a reduced exit from the BM.

Notch1 and Notch2 were correlated to different set of genes in our study suggesting that Notch1 was related to CD49a expression, IL21r, IL12rb1. Modifications in integrin expression are observed mainly in ILC1 with a clear decrease of surface CD49a. CD49a is not changed in Notch2-deficient mice arguing that modulation of CD49a levels is a specific Notch1 related feature. Hence, different Notch receptors could have different repercussions on the resulting subset especially if they are expressed at different frequencies. To consider whether Notch signaling could directly act on CD49a expression, we looked for potential binding sites for RBPJ in the promoter region of the itga1 gene (Figure S8 in Supplementary Material). Typical RBPJ-binding sequence (Figure S8A in Supplementary Material) was searched by screening the itga1 promoter region for a minimum of 5-mer motif. Three potential-binding sites were found suggesting a possible direct action of the Notch signaling pathway on the level of CD49a expression (Figure S8B in Supplementary Material). Due to the important proportion of type 1 ILC expressing Notch2 in the periphery and the superposition of the effects driven by RBPJ and Notch2 deficiency on enhanced effector functions, we suggest that Notch2 could be the main player in cell activation. Notch2 has been proposed as a central receptor for peripheral T cell maturation (34, 35). However, Notch1 could also control Eomes, perforin, and GzmB (36). Among others, Notch2 gene expression is correlated with the upregulation of Eomes, GzmB Cx3cr1, Ly6c, Il18r1, and IL2ra expression that constitute a hallmark of activated mature cNK cells. Increased expression of CX3CR1 was also described in T-bet-deficient mice (26) and is a marker of circulating peripheral cNK cells (37). Increased of Th1 cytokine receptors coupled with the increase of GzmB correspond to the phenotype of peripheral-activated cytotoxic cells. In our RBPJ-deficient model, type 1 ILC also increases their capacity to release IFNg and TNFa. The increase of Ly6C by RBPJ-deficient cNK cells is reminiscent of cells previously designated as peripheral resting inert mNK cells that could produce an effective and strong response in case of reactivation by cytokine stimuli (38). We suggest that maturation to the Ly6C^hi^ stage is linked to modification of T-bet/Eomes quantities driven by a deficiency in Notch signaling.

It was shown that Notch signaling impacts Th1 differentiation and cytotoxicity (34, 35, 39, 40). We found the opposite effect in type 1 ILC where the absence of the Notch signaling induces maturation of peripheral subsets toward a more “activated” state with enhanced cytotoxic functions. Nonetheless, our results are in agreement with other studies showing the maintenance of the Th1 response in Notch1/Notch2, RBPBJ-deficient, and MAML1 dominant-negative mice (41, 42).

Type 1 helper innate lymphoid cells and cNK cells are developmentally and functionally related and it has been suggested that cells can interconvert in certain conditions such as in a tumoral environment (25). In addition, dysregulations of the Notch signaling were described in diverse types of cancer where an oncogenic or tumor suppressive role depended on tissue type and particular microenvironments (43). In hepatocellular carcinoma, the implication of Notch signaling is currently under intense investigation (44).

To test antitumor ability of Notch-deficient cNK subsets, we used an *in vivo* hepatocellular carcinoma model. Hepa1–6 tumors grew slower at early phase when transplanted into Notch-deficient mice than into Notch-competent littermates thanks to an increase of intratumoral type 1 ILC frequency in RBPJ-deficient conditions. Moreover, deficient Notch signaling pathway leads to the presence of new CD49a^+^ cNK cells with a higher ability to release cytotoxic and inflammatory signals. Since in Notch-deficient mice, T cell subsets are reduced, this model is not ideal to compare later stages of tumor progression or regression. The progression of tumor area and analyses of intratumoral immune content in control animals allowed us to determine that tumors start to regress 2 weeks after hepatocellular carcinoma injections due to intratumoral effector T cells. The tumor regression is fast with already half of control littermates that have totally eradicated the tumor 3 weeks after injection. Hence, we decided to transfer T cells to both Notch-deficient and littermate controls to compare the tumor growth and analyze their immune compartment. As previously observed for early phase, RBPJ-deficient conditions allowed a better control of tumor growth until 10 days. To recover enough immune cells from the tumor, we did not extend our analysis over 2 weeks and observed that even after T cell transfer, type 1 ILC were more frequent and more cytotoxic in RBPJ-deficient animals. We concluded that inhibition of the Notch signaling pathway is beneficial for the early control of tumor growth by type 1 ILC. Other studies have shown that depending on Notch receptor and ligands identity, antagonistic effects could be found on tumor progression (45). Our study adds a stone by dissecting the regulation of important cytotoxic subsets implicated in the tumor immunosurveillance. Collectively, our data suggest that cNK cells unable to signal *via* the Notch pathway are more critical effector cells to restrain early carcinoma growth. These cells displayed features that resemble ILC1 but also mature reactivated cNK secreting higher amounts of cytotoxic and inflammatory cytokines. Tumor immunosurveillance studies using T-bet-deficient mice demonstrated that T-bet is essentially required at late stages of the immune response but is not crucial in primary tumors (26, 46, 47). Nonetheless, forced expression of Eomes in cNK cells was shown to decrease tumor growth and enhance survival (48). Hence, we propose that the Notch pathway in mature peripheral type 1 ILC represents a modulator of the inflammatory response. This is achieved by the regulation of T-bet versus Eomes expression and by regulating expression of pro/anti-apoptotic molecules, as observed on our comparative transcriptomic analyses. The Notch signaling pathway is also implicated in the regulation of the cNK cell-mediated tumor immunosurveillance. Hence, the tumoral environment could also temper the immune response *via* the regulation of Notch ligand expression.

Finally, we propose that the Notch signaling pathway as one of the extrinsic signals that control the intrinsic T-bet/Eomes balance. This pathway is implicated at multiple levels since T-bet/Eomes ratio are so important for cytotoxic lymphocyte differentiation and functions (49). Like TGF-β signaling that directs differentiation of salivary gland ILC1 through suppression of Eomes (25), we propose that the Notch signaling pathway participates to reduce Eomes levels in both cNK and ILC1, with a strong effect on hepatic ILC1. The spatio-temporal regulation of Notch receptor expression is participating to this equilibrium and enhances the complexity of the global picture.

## Ethics Statement

Mice were bred in accordance with Pasteur Institute guidelines in compliance with European animal welfare regulations, and all animal studies were approved by Pasteur Institute Safety Committee in accordance with French and European guidelines.

## Author Contributions

TP, SM, MP, and E-GB performed the experiments. RG, TP, and SM designed the experiments. RG, TP, AC, MP, SM, and E-GB analyzed the data. RG supervised the experiments and wrote the manuscript with the contribution of AC and TP.

## Conflict of Interest Statement

The authors declare that the research was conducted in the absence of any commercial or financial relationships that could be construed as a potential conflict of interest.

## Supplementary Material

Figure S1 **(A)** Correlation heatmap of gene expression from transcripts of Figure 1. **G** using Spearman method. **(B)** Correlation heatmap of gene expression from transcripts of signature 1 from Figure 1. **G** using Spearman method. **(C)** Correlation heatmap of gene expression from transcripts of signature 2 from Figure 1. **G** using Spearman method. Levels of correlation are shown from blue (low level) to red (high level).

Figure S2 **(A)** Flow cytometry of hepatic group 1 innate lymphoid cell in IL7r^Cre^ Notch2^F/F^, Vav^Cre^ Rbpj^F/F^ mice and their respective controls. **(B)** Mean fluorescence intensity of CD49a and CD49b in hepatic conventional NK (cNK) and type 1 helper innate lymphoid cells (ILC1) in control (white) and Vav^Cre^ Rbpj^F/F^ (red) mice. **(C)** Frequency of hepatic ILC1 and cNK in IL7r^Cre^ Notch2^F/F^, Vav^Cre^ Rbpj^F/F^ mice, and their respective controls.

Figure S3 **(A)** Frequency of hepatic type 1 helper innate lymphoid cells (ILC1) and conventional NK (cNK) in G0 (white), G1 (gray), and S/G2/M (black) phase. **(B)** T cells in the thymus were used as control of cell cycle.

Figure S4 Expression of TNFa, IFNg, and granzyme B in hepatic type 1 helper innate lymphoid cells (ILC1) (left panel) and conventional NK (cNK) (right panel) of control IL7r^Cre^ Notch2^F/+^ mice (top panel) and IL7r^Cre^ Notch2^F/F^ mice (middle panel). Levels of expression were compared (bottom panel) between control IL7r^Cre^ Notch2^F/+^ mice (blue) and IL7r^Cre^ Notch2^F/F^ mice (red), Lineage-negative cells were used as control for expression (dashed black).

Figure S5 Correlation heatmap of gene expression using Spearman method. Levels of correlation are shown from blue (low level) to red (high level).

Figure S6 **(A)** Flow cytometry of T cell infiltrate from tumor at day 14. Intracellular granzyme B (GzmB), TNFa, and IFNg expression of T cells. **(B)** Frequency of T cells infiltrate in alive CD45^+^ cells and frequency of T cells expressing GzmB, TNFa, and IFNg in alive CD45^+^ cells.

Figure S7 NK cells and NK progenitors (NKP) repartition in bone marrow (BM). **(A)** Flow cytometry of NKP (NKp46^−^ NK1.1^+^ CD49b^+/−^), and NK cells (NKp46^+^ NK1.1^+^) in BM of control IL7r^Cre^ Rbpj^F/+^ (top panel) and IL7r^Cre^ Rbpj^F/F^ mice (bottom panel). **(B)** Frequency of NKP (NKp46^−^ NK1.1^−^ CD49b^+/−^) and NK cells (NKp46^+^ NK1.1^+^) in BM of control IL7r^Cre^ Rbpj^F/+^ (white) and IL7r^Cre^ Rbpj^F/F^ mice (red). NKP were divided based on CD49b expression and NK cells were divided based on CD27 and Mac1 expression.

Figure S8 **(A)** Consensus sequence for RBPJ-binding sites to promoter regions. **(B)** Location of 5-mer motifs for potential RBPJ binding sites along the itga1 (CD49a) promoter region. Different motifs are represented in different colors.
