# Supplementary figures and images for: The Notch Signaling Pathway Is Balancing Type 1 Innate Lymphoid Cell Immune Functions
Source: Front Immunol. 2018 Jun 7;9:1252. doi: 10.3389/fimmu.2018.01252 (PMC5999736; doi:10.3389/fimmu.2018.01252)

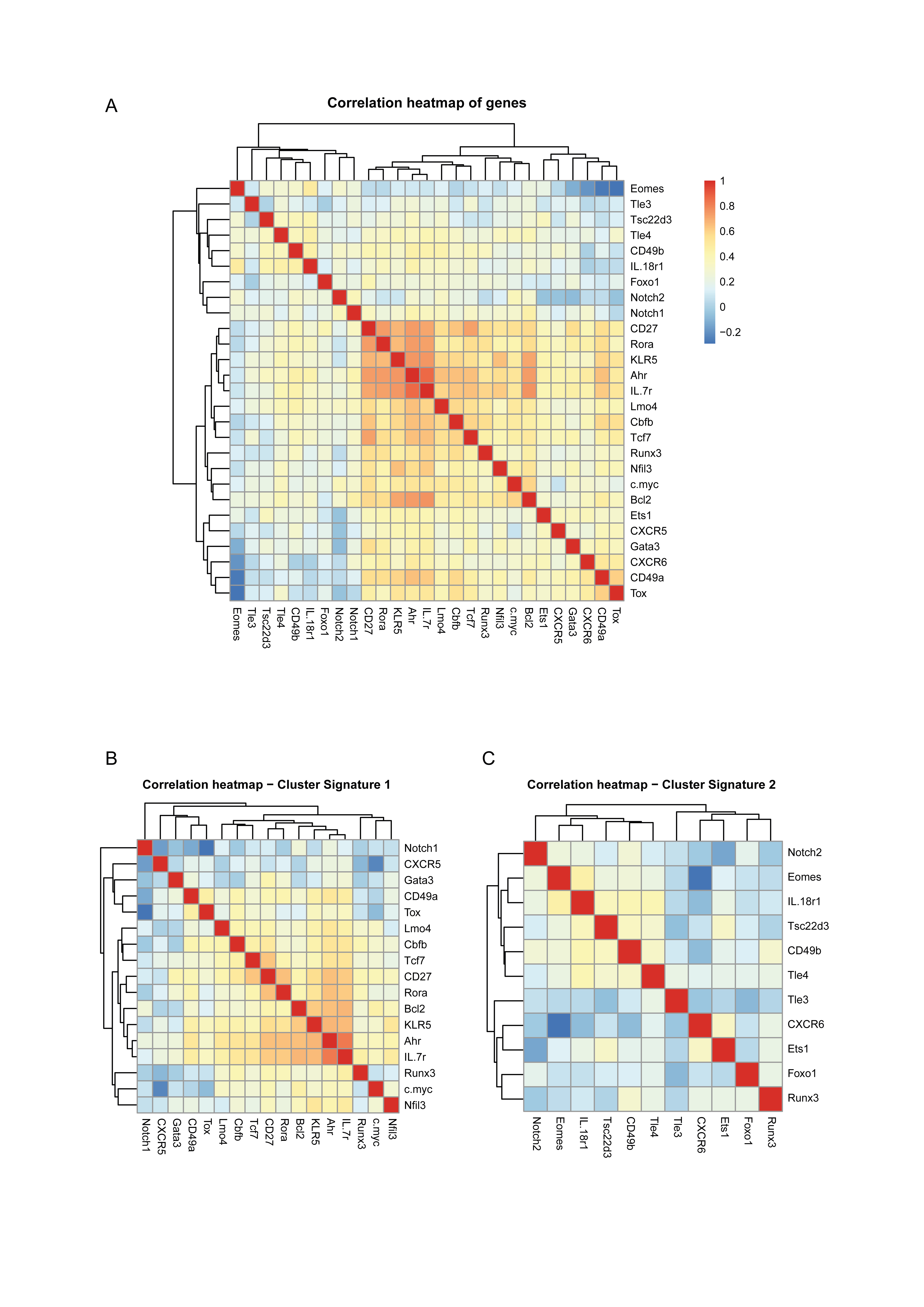

Supplement: Figure S1 — (A) Correlation heatmap of gene expression from transcripts of Figure 1. G using Spearman method. (B) Correlation heatmap of gene expression from transcripts of signature 1 from Figure 1. G using Spearman method. (C) Correlation heatmap of gene expression from transcripts of signature 2 from Figure 1. G using Spearman method. Levels of correlation are shown from blue (low level) to red (high level). [file Image_1.tif]

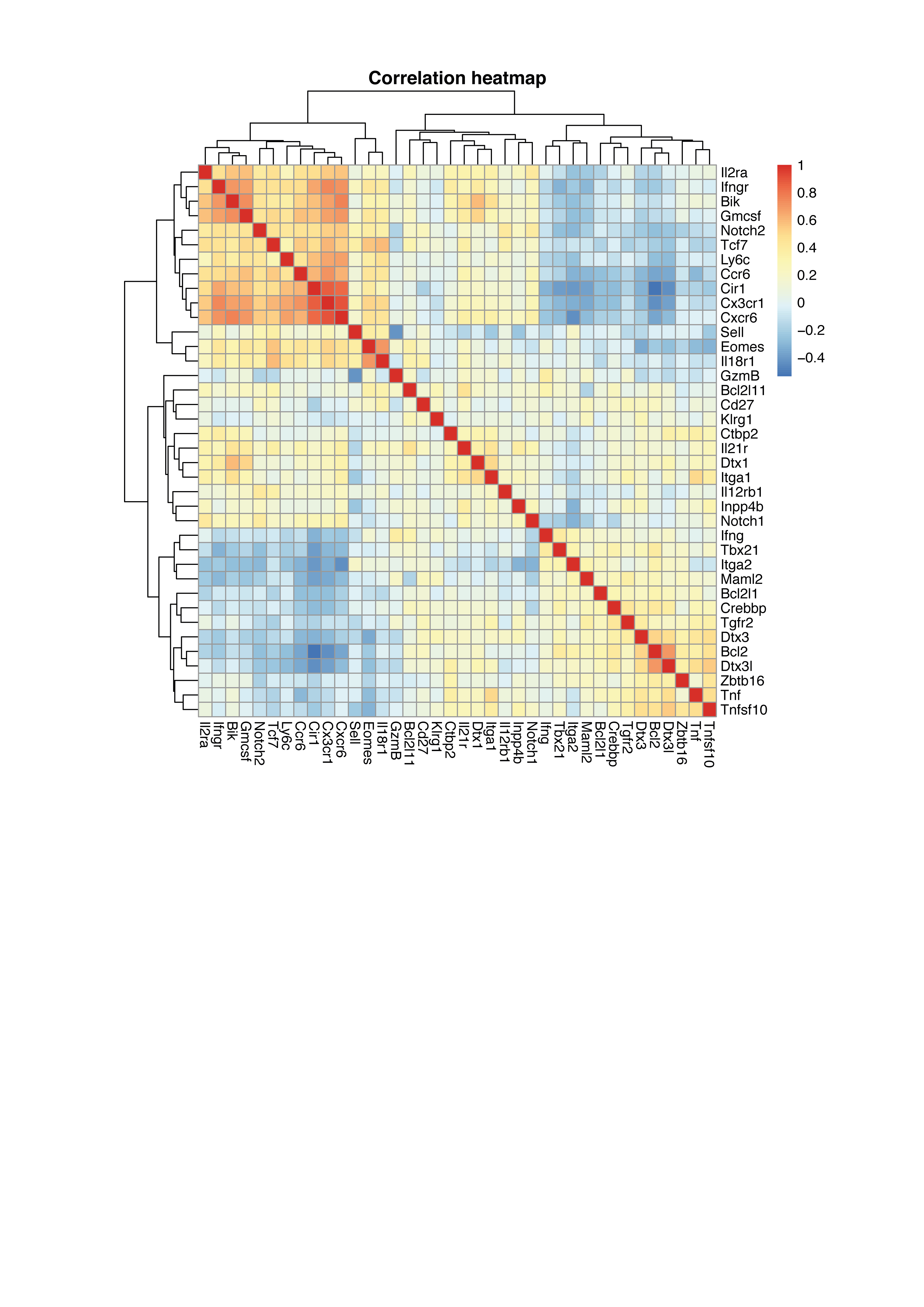

Supplement: Figure S5 — Correlation heatmap of gene expression using Spearman method. Levels of correlation are shown from blue (low level) to red (high level). [file Image_5.tif]
